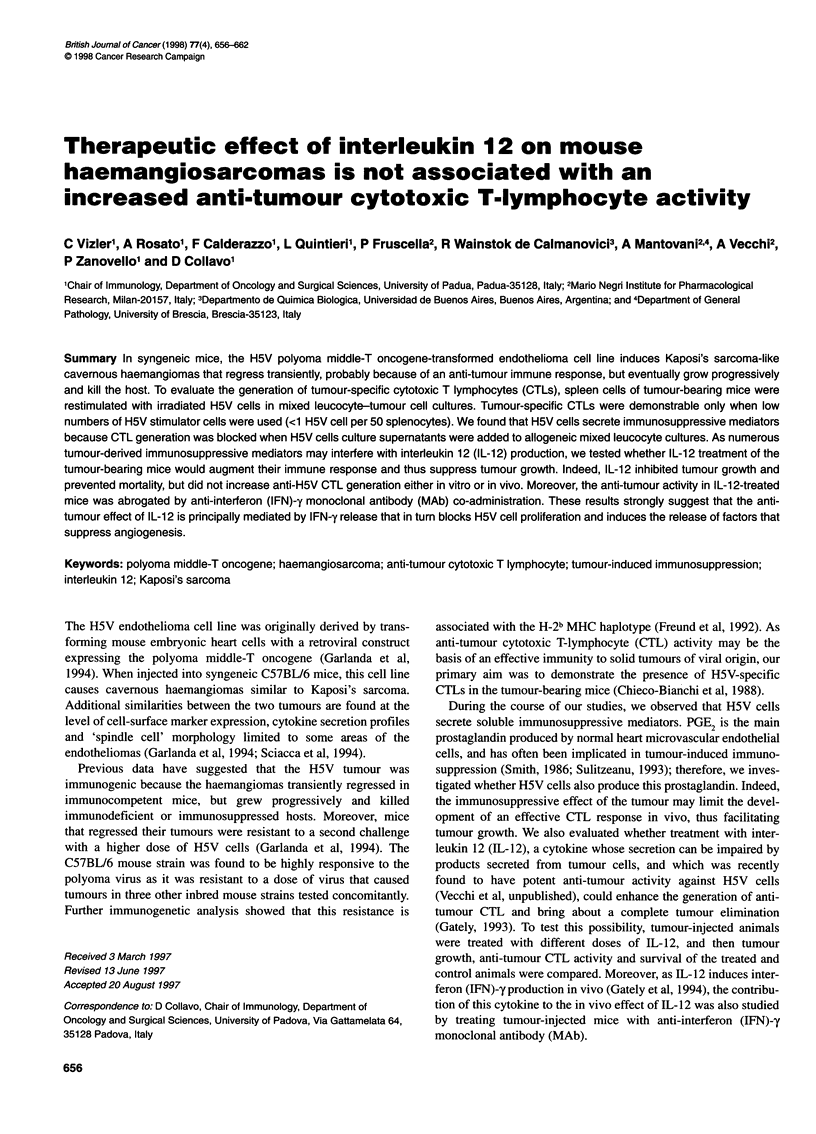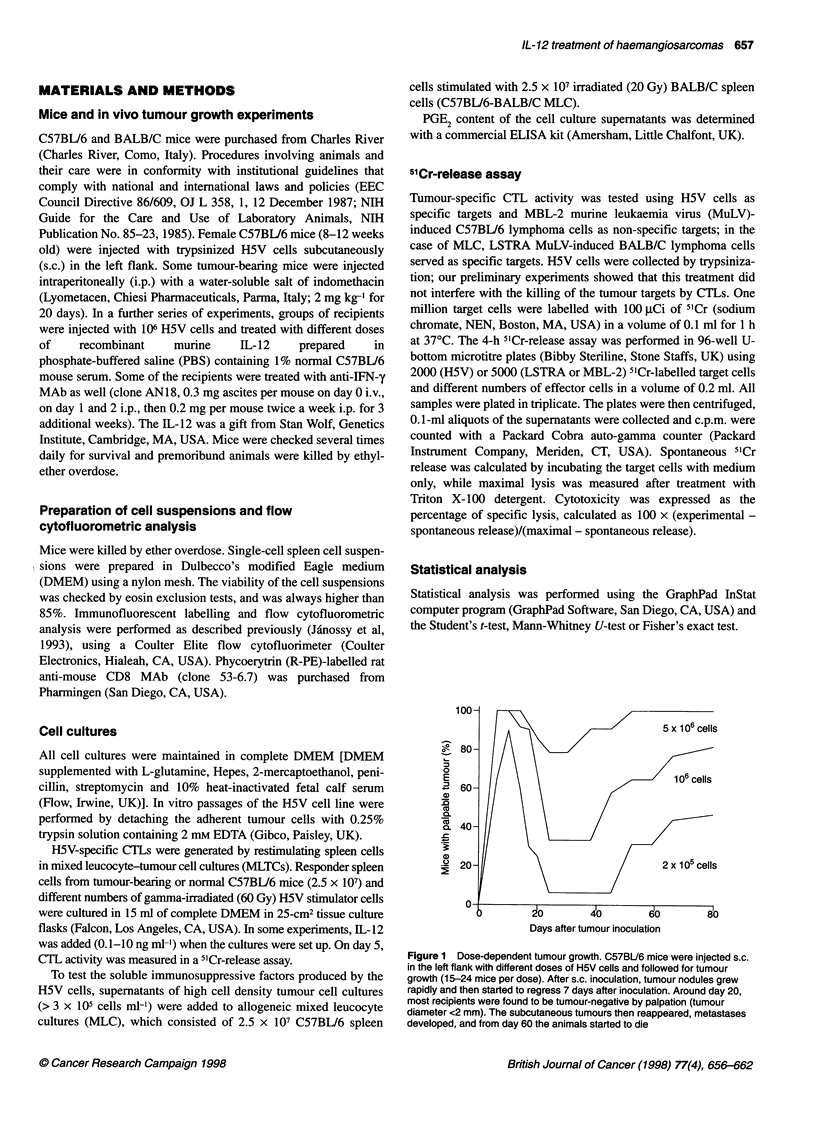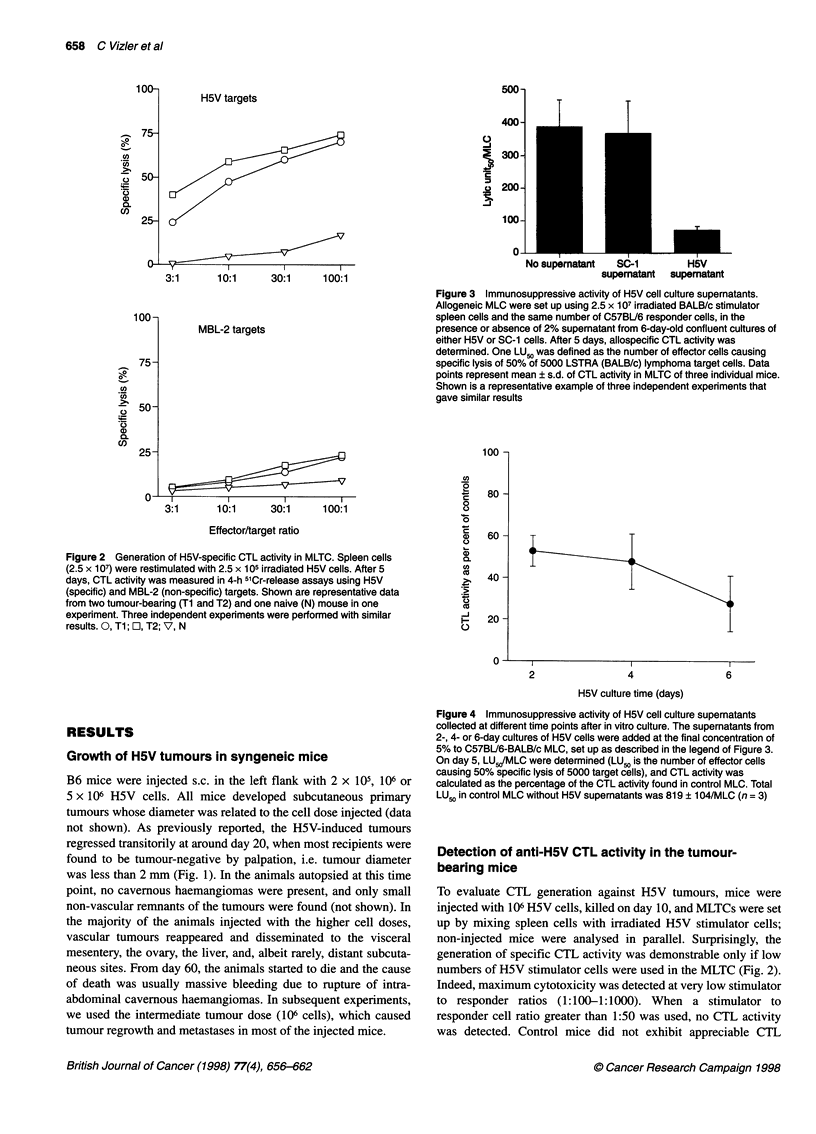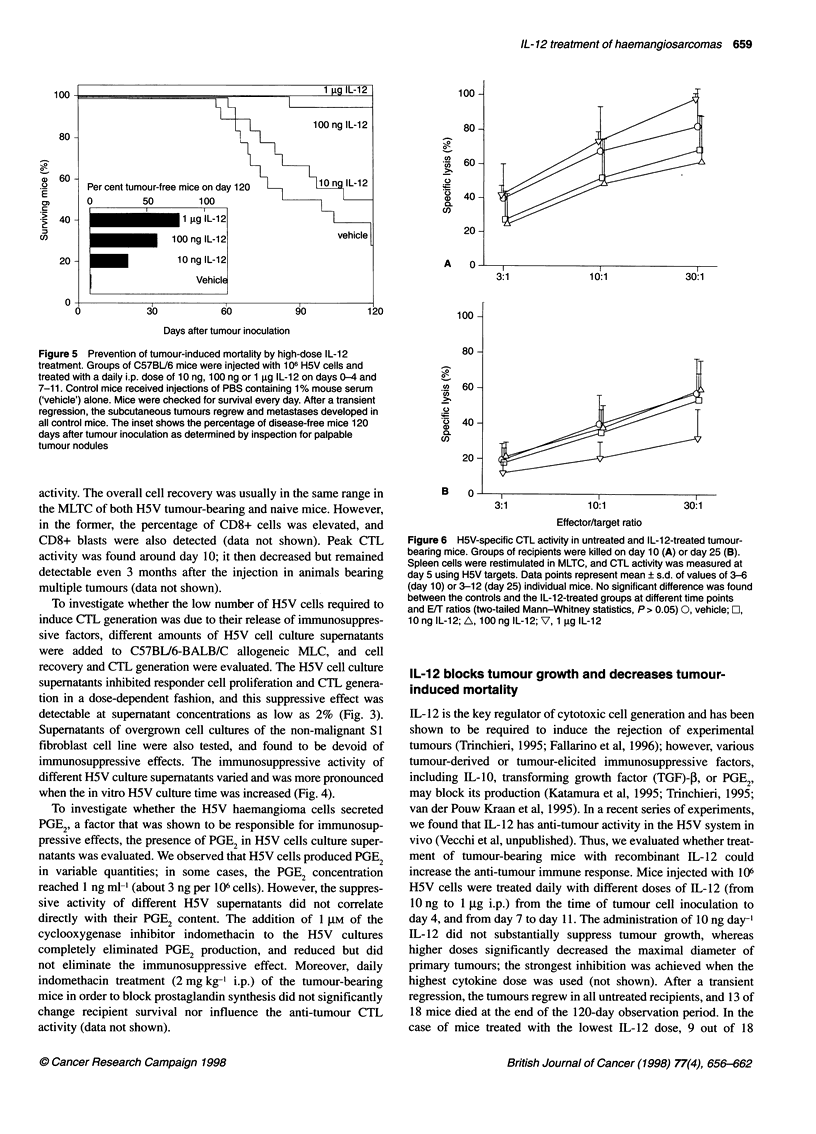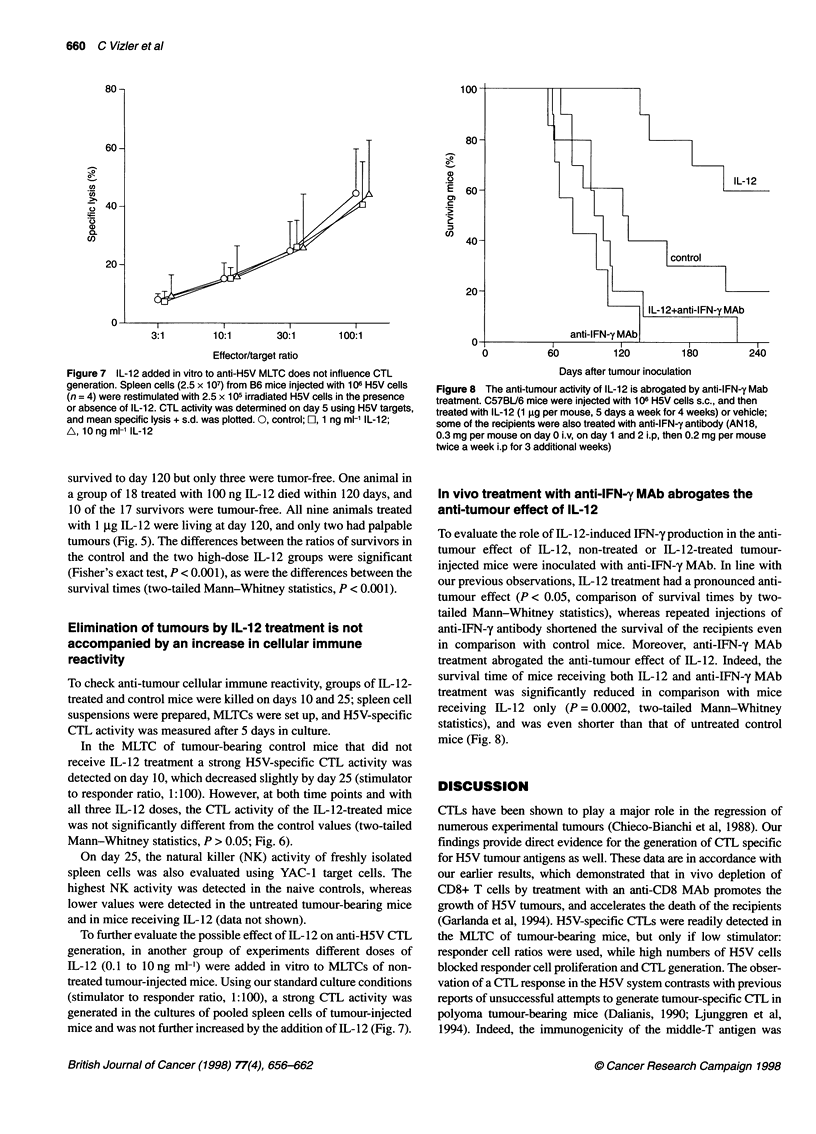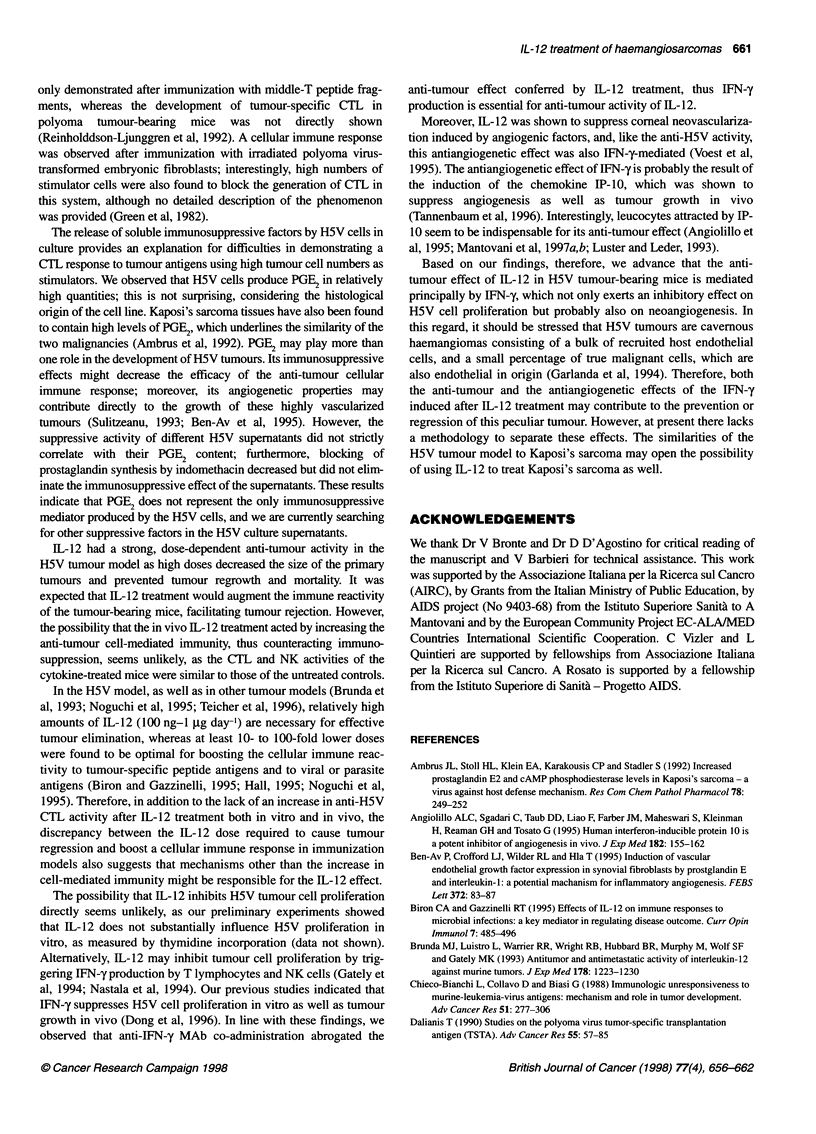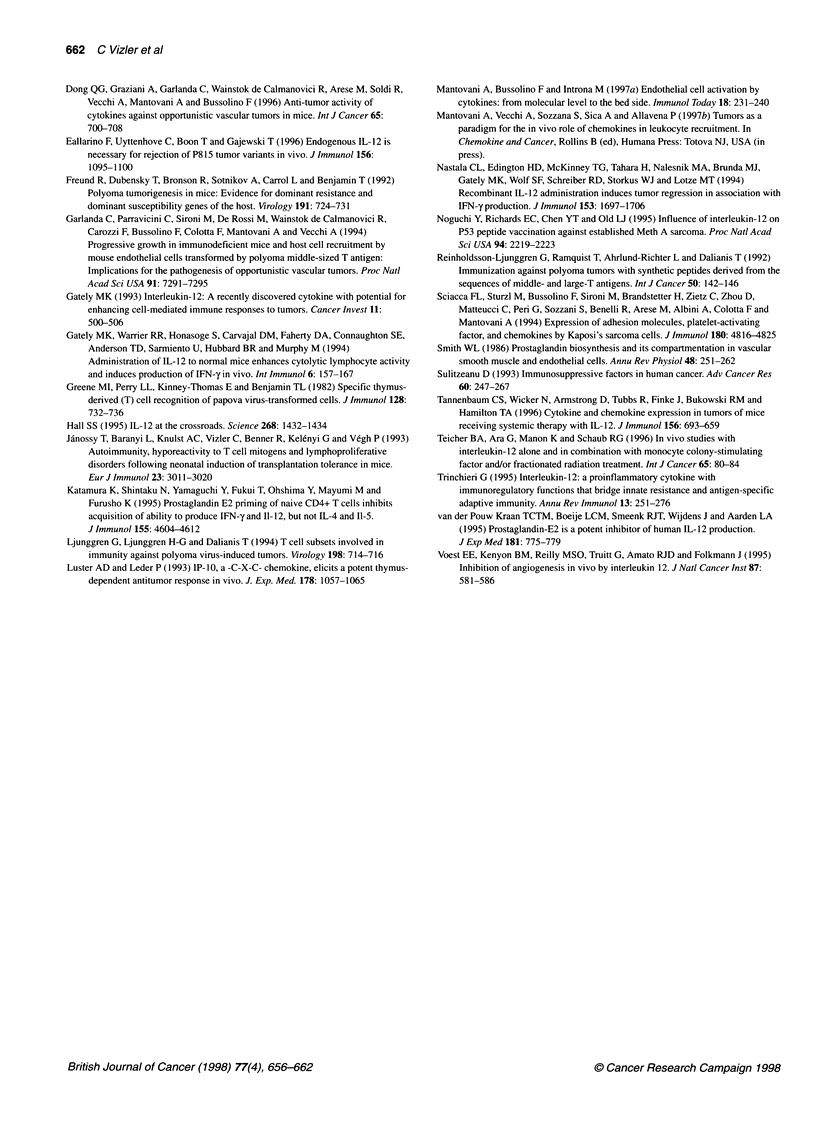# Therapeutic effect of interleukin 12 on mouse haemangiosarcomas is not associated with an increased anti-tumour cytotoxic T-lymphocyte activity.

**DOI:** 10.1038/bjc.1998.105

**Published:** 1998-02

**Authors:** C. Vizler, A. Rosato, F. Calderazzo, L. Quintieri, P. Fruscella, R. Wainstok de Calmanovici, A. Mantovani, A. Vecchi, P. Zanovello, D. Collavo

**Affiliations:** Department of Oncology and Surgical Sciences, University of Padua, Italy.

## Abstract

In syngeneic mice, the H5V polyoma middle-T oncogene-transformed endothelioma cell line induces Kaposi's sarcoma-like cavernous haemangiomas that regress transiently, probably because of an anti-tumour immune response, but eventually grow progressively and kill the host. To evaluate the generation of tumour-specific cytotoxic T lymphocytes (CTLs), spleen cells of tumour-bearing mice were restimulated with irradiated H5V cells in mixed leucocyte-tumour cell cultures. Tumour-specific CTLs were demonstrable only when low numbers of H5V stimulator cells were used (<1 H5V cell per 50 splenocytes). We found that H5V cells secrete immunosuppressive mediators because CTL generation was blocked when H5V cells culture supernatants were added to allogeneic mixed leucocyte cultures. As numerous tumour-derived immunosuppressive mediators may interfere with interleukin 12 (IL-12) production, we tested whether IL-12 treatment of the tumour-bearing mice would augment their immune response and thus suppress tumour growth. Indeed, IL-12 inhibited tumour growth and prevented mortality, but did not increase anti-H5V CTL generation either in vitro or in vivo. Moreover, the anti-tumour activity in IL-12-treated mice was abrogated by anti-interferon (IFN)-gamma monoclonal antibody (MAb) co-administration. These results strongly suggest that the anti-tumour effect of IL-12 is principally mediated by IFN-gamma release that in turn blocks H5V cell proliferation and induces the release of factors that suppress angiogenesis.


British Journal of Cancer (1998) 77(4), 656–662
© 1998 Cancer Research Campaign

### Therapeutic effect of interleukin 12 on mouse haemangiosarcomas is not associated with an increased anti-tumour cytotoxic T-lymphocyte activity

**C Vizler[1], A Rosato[1], F Calderazzo[1], L Quintieri[1], P Fruscella[2], R Wainstok de Calmanovici[3], A Mantovani[2,4], A Vecchi[2], P Zanovello[1] and D Collavo[1]**

[1]Chair of Immunology, Department of Oncology and Surgical Sciences, University of Padua, Padua-35128, Italy; [2]Mario Negri Institute for Pharmacological Research, Milan-20157, Italy; [3]Departmento de Quimica Biologica, Universidad de Buenos Aires, Buenos Aires, Argentina; and [4]Department of General Pathology, University of Brescia, Brescia-35123, Italy

**Summary** In syngeneic mice, the H5V polyoma middle-T oncogene-transformed endothelioma cell line induces Kaposi's sarcoma-like cavernous haemangiomas that regress transiently, probably because of an anti-tumour immune response, but eventually grow progressively and kill the host. To evaluate the generation of tumour-specific cytotoxic T lymphocytes (CTLs), spleen cells of tumour-bearing mice were restimulated with irradiated H5V cells in mixed leucocyte–tumour cell cultures. Tumour-specific CTLs were demonstrable only when low numbers of H5V stimulator cells were used (<1 H5V cell per 50 splenocytes). We found that H5V cells secrete immunosuppressive mediators because CTL generation was blocked when H5V cells culture supernatants were added to allogeneic mixed leucocyte cultures. As numerous tumour-derived immunosuppressive mediators may interfere with interleukin 12 (IL-12) production, we tested whether IL-12 treatment of the tumour-bearing mice would augment their immune response and thus suppress tumour growth. Indeed, IL-12 inhibited tumour growth and prevented mortality, but did not increase anti-H5V CTL generation either in vitro or in vivo. Moreover, the anti-tumour activity in IL-12-treated mice was abrogated by anti-interferon (IFN)-γ monoclonal antibody (MAb) co-administration. These results strongly suggest that the anti-tumour effect of IL-12 is principally mediated by IFN-γ release that in turn blocks H5V cell proliferation and induces the release of factors that suppress angiogenesis.

**Keywords:** polyoma middle-T oncogene; haemangiosarcoma; anti-tumour cytotoxic T lymphocyte; tumour-induced immunosuppression; interleukin 12; Kaposi's sarcoma

The H5V endothelioma cell line was originally derived by transforming mouse embryonic heart cells with a retroviral construct expressing the polyoma middle-T oncogene (Garlanda et al, 1994). When injected into syngeneic C57BL/6 mice, this cell line causes cavernous haemangiomas similar to Kaposi's sarcoma. Additional similarities between the two tumours are found at the level of cell-surface marker expression, cytokine secretion profiles and 'spindle cell' morphology limited to some areas of the endotheliomas (Garlanda et al, 1994; Sciacca et al, 1994).

Previous data have suggested that the H5V tumour was immunogenic because the haemangiomas transiently regressed in immunocompetent mice, but grew progressively and killed immunodeficient or immunosuppressed hosts. Moreover, mice that regressed their tumours were resistant to a second challenge with a higher dose of H5V cells (Garlanda et al, 1994). The C57BL/6 mouse strain was found to be highly responsive to the polyoma virus as it was resistant to a dose of virus that caused tumours in three other inbred mouse strains tested concomitantly. Further immunogenetic analysis showed that this resistance is associated with the H-$2^b$ MHC haplotype (Freund et al, 1992). As anti-tumour cytotoxic T-lymphocyte (CTL) activity may be the basis of an effective immunity to solid tumours of viral origin, our primary aim was to demonstrate the presence of H5V-specific CTLs in the tumour-bearing mice (Chieco-Bianchi et al, 1988).

During the course of our studies, we observed that H5V cells secrete soluble immunosuppressive mediators. $PGE_2$ is the main prostaglandin produced by normal heart microvascular endothelial cells, and has often been implicated in tumour-induced immunosuppression (Smith, 1986; Sulitzeanu, 1993); therefore, we investigated whether H5V cells also produce this prostaglandin. Indeed, the immunosuppressive effect of the tumour may limit the development of an effective CTL response in vivo, thus facilitating tumour growth. We also evaluated whether treatment with interleukin 12 (IL-12), a cytokine whose secretion can be impaired by products secreted from tumour cells, and which was recently found to have potent anti-tumour activity against H5V cells (Vecchi et al, unpublished), could enhance the generation of anti-tumour CTL and bring about a complete tumour elimination (Gately, 1993). To test this possibility, tumour-injected animals were treated with different doses of IL-12, and then tumour growth, anti-tumour CTL activity and survival of the treated and control animals were compared. Moreover, as IL-12 induces interferon (IFN)-γ production in vivo (Gately et al, 1994), the contribution of this cytokine to the in vivo effect of IL-12 was also studied by treating tumour-injected mice with anti-interferon (IFN)-γ monoclonal antibody (MAb).

*Received 3 March 1997*
*Revised 13 June 1997*
*Accepted 20 August 1997*

*Correspondence to:* D Collavo, Chair of Immunology, Department of Oncology and Surgical Sciences, University of Padova, Via Gattamelata 64, 35128 Padova, Italy

### MATERIALS AND METHODS

### Mice and in vivo tumour growth experiments

C57BL/6 and BALB/C mice were purchased from Charles River (Charles River, Como, Italy). Procedures involving animals and their care were in conformity with institutional guidelines that comply with national and international laws and policies (EEC Council Directive 86/609, OJ L 358, 1, 12 December 1987; NIH Guide for the Care and Use of Laboratory Animals, NIH Publication No. 85–23, 1985). Female C57BL/6 mice (8–12 weeks old) were injected with trypsinized H5V cells subcutaneously (s.c.) in the left flank. Some tumour-bearing mice were injected intraperitoneally (i.p.) with a water-soluble salt of indomethacin (Lyometacen, Chiesi Pharmaceuticals, Parma, Italy; 2 mg $kg^{-1}$ for 20 days). In a further series of experiments, groups of recipients were injected with $10^6$ H5V cells and treated with different doses of recombinant murine IL-12 prepared in phosphate-buffered saline (PBS) containing 1% normal C57BL/6 mouse serum. Some of the recipients were treated with anti-IFN-$\gamma$ MAb as well (clone AN18, 0.3 mg ascites per mouse on day 0 i.v., on day 1 and 2 i.p., then 0.2 mg per mouse twice a week i.p. for 3 additional weeks). The IL-12 was a gift from Stan Wolf, Genetics Institute, Cambridge, MA, USA. Mice were checked several times daily for survival and premoribund animals were killed by ethyl-ether overdose.

### Preparation of cell suspensions and flow cytofluorometric analysis

Mice were killed by ether overdose. Single-cell spleen cell suspensions were prepared in Dulbecco's modified Eagle medium (DMEM) using a nylon mesh. The viability of the cell suspensions was checked by eosin exclusion tests, and was always higher than 85%. Immunofluorescent labelling and flow cytofluorometric analysis were performed as described previously (Jánossy et al, 1993), using a Coulter Elite flow cytofluorimeter (Coulter Electronics, Hialeah, CA, USA). Phycoerytrin (R-PE)-labelled rat anti-mouse CD8 MAb (clone 53-6.7) was purchased from Pharmingen (San Diego, CA, USA).

### Cell cultures

All cell cultures were maintained in complete DMEM [DMEM supplemented with L-glutamine, Hepes, 2-mercaptoethanol, penicillin, streptomycin and 10% heat-inactivated fetal calf serum (Flow, Irwine, UK)]. In vitro passages of the H5V cell line were performed by detaching the adherent tumour cells with 0.25% trypsin solution containing 2 mM EDTA (Gibco, Paisley, UK).

H5V-specific CTLs were generated by restimulating spleen cells in mixed leucocyte–tumour cell cultures (MLTCs). Responder spleen cells from tumour-bearing or normal C57BL/6 mice ($2.5 \times 10^7$) and different numbers of gamma-irradiated (60 Gy) H5V stimulator cells were cultured in 15 ml of complete DMEM in 25-$cm^2$ tissue culture flasks (Falcon, Los Angeles, CA, USA). In some experiments, IL-12 was added (0.1–10 ng $ml^{-1}$) when the cultures were set up. On day 5, CTL activity was measured in a $^{51}Cr$-release assay.

To test the soluble immunosuppressive factors produced by the H5V cells, supernatants of high cell density tumour cell cultures ($> 3 \times 10^5$ cells $ml^{-1}$) were added to allogeneic mixed leucocyte cultures (MLC), which consisted of $2.5 \times 10^7$ C57BL/6 spleen cells stimulated with $2.5 \times 10^7$ irradiated (20 Gy) BALB/C spleen cells (C57BL/6-BALB/C MLC).

$PGE_2$ content of the cell culture supernatants was determined with a commercial ELISA kit (Amersham, Little Chalfont, UK).

### $^{51}Cr$-release assay

Tumour-specific CTL activity was tested using H5V cells as specific targets and MBL-2 murine leukaemia virus (MuLV)-induced C57BL/6 lymphoma cells as non-specific targets; in the case of MLC, LSTRA MuLV-induced BALB/C lymphoma cells served as specific targets. H5V cells were collected by trypsinization; our preliminary experiments showed that this treatment did not interfere with the killing of the tumour targets by CTLs. One million target cells were labelled with 100 µCi of $^{51}Cr$ (sodium chromate, NEN, Boston, MA, USA) in a volume of 0.1 ml for 1 h at 37°C. The 4-h $^{51}Cr$-release assay was performed in 96-well U-bottom microtitre plates (Bibby Steriline, Stone Staffs, UK) using 2000 (H5V) or 5000 (LSTRA or MBL-2) $^{51}Cr$-labelled target cells and different numbers of effector cells in a volume of 0.2 ml. All samples were plated in triplicate. The plates were then centrifuged, 0.1-ml aliquots of the supernatants were collected and c.p.m. were counted with a Packard Cobra auto-gamma counter (Packard Instrument Company, Meriden, CT, USA). Spontaneous $^{51}Cr$ release was calculated by incubating the target cells with medium only, while maximal lysis was measured after treatment with Triton X-100 detergent. Cytotoxicity was expressed as the percentage of specific lysis, calculated as 100 × (experimental – spontaneous release)/(maximal – spontaneous release).

### Statistical analysis

Statistical analysis was performed using the GraphPad InStat computer program (GraphPad Software, San Diego, CA, USA) and the Student's *t*-test, Mann-Whitney *U*-test or Fisher's exact test.

**Figure 1** Dose-dependent tumour growth. C57BL/6 mice were injected s.c. in the left flank with different doses of H5V cells and followed for tumour growth (15–24 mice per dose). After s.c. inoculation, tumour nodules grew rapidly and then started to regress 7 days after inoculation. Around day 20, most recipients were found to be tumour-negative by palpation (tumour diameter <2 mm). The subcutaneous tumours then reappeared, metastases developed, and from day 60 the animals started to die

© Cancer Research Campaign 1998

**Figure 2** Generation of H5V-specific CTL activity in MLTC. Spleen cells (2.5 x $10^7$) were restimulated with 2.5 x $10^5$ irradiated H5V cells. After 5 days, CTL activity was measured in 4-h $^{51}$Cr-release assays using H5V (specific) and MBL-2 (non-specific) targets. Shown are representative data from two tumour-bearing (T1 and T2) and one naive (N) mouse in one experiment. Three independent experiments were performed with similar results. ○, T1; □, T2; ▽, N

**Figure 3** Immunosuppressive activity of H5V cell culture supernatants. Allogeneic MLC were set up using 2.5 x $10^7$ irradiated BALB/c stimulator spleen cells and the same number of C57BL/6 responder cells, in the presence or absence of 2% supernatant from 6-day-old confluent cultures of either H5V or SC-1 cells. After 5 days, allospecific CTL activity was determined. One $LU_{50}$ was defined as the number of effector cells causing specific lysis of 50% of 5000 LSTRA (BALB/c) lymphoma target cells. Data points represent mean ± s.d. of CTL activity in MLTC of three individual mice. Shown is a representative example of three independent experiments that gave similar results

**Figure 4** Immunosuppressive activity of H5V cell culture supernatants collected at different time points after in vitro culture. The supernatants from 2-, 4- or 6-day cultures of H5V cells were added at the final concentration of 5% to C57BL/6-BALB/c MLC, set up as described in the legend of Figure 3. On day 5, $LU_{50}$/MLC were determined ($LU_{50}$ is the number of effector cells causing 50% specific lysis of 5000 target cells), and CTL activity was calculated as the percentage of the CTL activity found in control MLC. Total $LU_{50}$ in control MLC without H5V supernatants was 819 ± 104/MLC ($n$ = 3)

### RESULTS

### Growth of H5V tumours in syngeneic mice

B6 mice were injected s.c. in the left flank with 2 × $10^5$, $10^6$ or 5 × $10^6$ H5V cells. All mice developed subcutaneous primary tumours whose diameter was related to the cell dose injected (data not shown). As previously reported, the H5V-induced tumours regressed transitorily at around day 20, when most recipients were found to be tumour-negative by palpation, i.e. tumour diameter was less than 2 mm (Fig. 1). In the animals autopsied at this time point, no cavernous haemangiomas were present, and only small non-vascular remnants of the tumours were found (not shown). In the majority of the animals injected with the higher cell doses, vascular tumours reappeared and disseminated to the visceral mesentery, the ovary, the liver, and, albeit rarely, distant subcutaneous sites. From day 60, the animals started to die and the cause of death was usually massive bleeding due to rupture of intra-abdominal cavernous haemangiomas. In subsequent experiments, we used the intermediate tumour dose ($10^6$ cells), which caused tumour regrowth and metastases in most of the injected mice.

### Detection of anti-H5V CTL activity in the tumour-bearing mice

To evaluate CTL generation against H5V tumours, mice were injected with $10^6$ H5V cells, killed on day 10, and MLTCs were set up by mixing spleen cells with irradiated H5V stimulator cells; non-injected mice were analysed in parallel. Surprisingly, the generation of specific CTL activity was demonstrable only if low numbers of H5V stimulator cells were used in the MLTC (Fig. 2). Indeed, maximum cytotoxicity was detected at very low stimulator to responder ratios (1:100–1:1000). When a stimulator to responder cell ratio greater than 1:50 was used, no CTL activity was detected. Control mice did not exhibit appreciable CTL

© Cancer Research Campaign 1998

**Figure 5** Prevention of tumour-induced mortality by high-dose IL-12 treatment. Groups of C57BL/6 mice were injected with $10^6$ H5V cells and treated with a daily i.p. dose of 10 ng, 100 ng or 1 µg IL-12 on days 0–4 and 7–11. Control mice received injections of PBS containing 1% mouse serum ('vehicle') alone. Mice were checked for survival every day. After a transient regression, the subcutaneous tumours regrew and metastases developed in all control mice. The inset shows the percentage of disease-free mice 120 days after tumour inoculation as determined by inspection for palpable tumour nodules

**Figure 6** H5V-specific CTL activity in untreated and IL-12-treated tumour-bearing mice. Groups of recipients were killed on day 10 (**A**) or day 25 (**B**). Spleen cells were restimulated in MLTC, and CTL activity was measured at day 5 using H5V targets. Data points represent mean ± s.d. of values of 3–6 (day 10) or 3–12 (day 25) individual mice. No significant difference was found between the controls and the IL-12-treated groups at different time points and E/T ratios (two-tailed Mann–Whitney statistics, $P > 0.05$) ○, vehicle; □, 10 ng IL-12; △, 100 ng IL-12; ▽, 1 µg IL-12

activity. The overall cell recovery was usually in the same range in the MLTC of both H5V tumour-bearing and naive mice. However, in the former, the percentage of CD8+ cells was elevated, and CD8+ blasts were also detected (data not shown). Peak CTL activity was found around day 10; it then decreased but remained detectable even 3 months after the injection in animals bearing multiple tumours (data not shown).

To investigate whether the low number of H5V cells required to induce CTL generation was due to their release of immunosuppressive factors, different amounts of H5V cell culture supernatants were added to C57BL/6-BALB/C allogeneic MLC, and cell recovery and CTL generation were evaluated. The H5V cell culture supernatants inhibited responder cell proliferation and CTL generation in a dose-dependent fashion, and this suppressive effect was detectable at supernatant concentrations as low as 2% (Fig. 3). Supernatants of overgrown cell cultures of the non-malignant S1 fibroblast cell line were also tested, and found to be devoid of immunosuppressive effects. The immunosuppressive activity of different H5V culture supernatants varied and was more pronounced when the in vitro H5V culture time was increased (Fig. 4).

To investigate whether the H5V haemangioma cells secreted $PGE_2$, a factor that was shown to be responsible for immunosuppressive effects, the presence of $PGE_2$ in H5V cells culture supernatants was evaluated. We observed that H5V cells produced $PGE_2$ in variable quantities; in some cases, the $PGE_2$ concentration reached 1 ng $ml^{-1}$ (about 3 ng per $10^6$ cells). However, the suppressive activity of different H5V supernatants did not correlate directly with their $PGE_2$ content. The addition of 1 µM of the cyclooxygenase inhibitor indomethacin to the H5V cultures completely eliminated $PGE_2$ production, and reduced but did not eliminate the immunosuppressive effect. Moreover, daily indomethacin treatment (2 mg $kg^{-1}$ i.p.) of the tumour-bearing mice in order to block prostaglandin synthesis did not significantly change recipient survival nor influence the anti-tumour CTL activity (data not shown).

### IL-12 blocks tumour growth and decreases tumour-induced mortality

IL-12 is the key regulator of cytotoxic cell generation and has been shown to be required to induce the rejection of experimental tumours (Trinchieri, 1995; Fallarino et al, 1996); however, various tumour-derived or tumour-elicited immunosuppressive factors, including IL-10, transforming growth factor (TGF)-β, or $PGE_2$, may block its production (Katamura et al, 1995; Trinchieri, 1995; van der Pouw Kraan et al, 1995). In a recent series of experiments, we found that IL-12 has anti-tumour activity in the H5V system in vivo (Vecchi et al, unpublished). Thus, we evaluated whether treatment of tumour-bearing mice with recombinant IL-12 could increase the anti-tumour immune response. Mice injected with $10^6$ H5V cells were treated daily with different doses of IL-12 (from 10 ng to 1 µg i.p.) from the time of tumour cell inoculation to day 4, and from day 7 to day 11. The administration of 10 ng $day^{-1}$ IL-12 did not substantially suppress tumour growth, whereas higher doses significantly decreased the maximal diameter of primary tumours; the strongest inhibition was achieved when the highest cytokine dose was used (not shown). After a transient regression, the tumours regrew in all untreated recipients, and 13 of 18 mice died at the end of the 120-day observation period. In the case of mice treated with the lowest IL-12 dose, 9 out of 18

© Cancer Research Campaign 1998

**Figure 7** IL-12 added in vitro to anti-H5V MLTC does not influence CTL generation. Spleen cells ($2.5 \times 10^7$) from B6 mice injected with $10^6$ H5V cells ($n = 4$) were restimulated with $2.5 \times 10^5$ irradiated H5V cells in the presence or absence of IL-12. CTL activity was determined on day 5 using H5V targets, and mean specific lysis + s.d. was plotted. ○, control; □, 1 ng $ml^{-1}$ IL-12; △, 10 ng $ml^{-1}$ IL-12

**Figure 8** The anti-tumour activity of IL-12 is abrogated by anti-IFN-γ Mab treatment. C57BL/6 mice were injected with $10^6$ H5V cells s.c., and then treated with IL-12 (1 μg per mouse, 5 days a week for 4 weeks) or vehicle; some of the recipients were also treated with anti-IFN-γ antibody (AN18, 0.3 mg per mouse on day 0 i.v, on day 1 and 2 i.p, then 0.2 mg per mouse twice a week i.p for 3 additional weeks)

survived to day 120 but only three were tumor-free. One animal in a group of 18 treated with 100 ng IL-12 died within 120 days, and 10 of the 17 survivors were tumour-free. All nine animals treated with 1 μg IL-12 were living at day 120, and only two had palpable tumours (Fig. 5). The differences between the ratios of survivors in the control and the two high-dose IL-12 groups were significant (Fisher's exact test, $P < 0.001$), as were the differences between the survival times (two-tailed Mann–Whitney statistics, $P < 0.001$).

### Elimination of tumours by IL-12 treatment is not accompanied by an increase in cellular immune reactivity

To check anti-tumour cellular immune reactivity, groups of IL-12-treated and control mice were killed on days 10 and 25; spleen cell suspensions were prepared, MLTCs were set up, and H5V-specific CTL activity was measured after 5 days in culture.

In the MLTC of tumour-bearing control mice that did not receive IL-12 treatment a strong H5V-specific CTL activity was detected on day 10, which decreased slightly by day 25 (stimulator to responder ratio, 1:100). However, at both time points and with all three IL-12 doses, the CTL activity of the IL-12-treated mice was not significantly different from the control values (two-tailed Mann–Whitney statistics, $P > 0.05$; Fig. 6).

On day 25, the natural killer (NK) activity of freshly isolated spleen cells was also evaluated using YAC-1 target cells. The highest NK activity was detected in the naive controls, whereas lower values were detected in the untreated tumour-bearing mice and in mice receiving IL-12 (data not shown).

To further evaluate the possible effect of IL-12 on anti-H5V CTL generation, in another group of experiments different doses of IL-12 (0.1 to 10 ng $ml^{-1}$) were added in vitro to MLTCs of non-treated tumour-injected mice. Using our standard culture conditions (stimulator to responder ratio, 1:100), a strong CTL activity was generated in the cultures of pooled spleen cells of tumour-injected mice and was not further increased by the addition of IL-12 (Fig. 7).

### In vivo treatment with anti-IFN-γ MAb abrogates the anti-tumour effect of IL-12

To evaluate the role of IL-12-induced IFN-γ production in the anti-tumour effect of IL-12, non-treated or IL-12-treated tumour-injected mice were inoculated with anti-IFN-γ MAb. In line with our previous observations, IL-12 treatment had a pronounced anti-tumour effect ($P < 0.05$, comparison of survival times by two-tailed Mann–Whitney statistics), whereas repeated injections of anti-IFN-γ antibody shortened the survival of the recipients even in comparison with control mice. Moreover, anti-IFN-γ MAb treatment abrogated the anti-tumour effect of IL-12. Indeed, the survival time of mice receiving both IL-12 and anti-IFN-γ MAb treatment was significantly reduced in comparison with mice receiving IL-12 only ($P = 0.0002$, two-tailed Mann–Whitney statistics), and was even shorter than that of untreated control mice (Fig. 8).

### DISCUSSION

CTLs have been shown to play a major role in the regression of numerous experimental tumours (Chieco-Bianchi et al, 1988). Our findings provide direct evidence for the generation of CTL specific for H5V tumour antigens as well. These data are in accordance with our earlier results, which demonstrated that in vivo depletion of CD8+ T cells by treatment with an anti-CD8 MAb promotes the growth of H5V tumours, and accelerates the death of the recipients (Garlanda et al, 1994). H5V-specific CTLs were readily detected in the MLTC of tumour-bearing mice, but only if low stimulator: responder cell ratios were used, while high numbers of H5V cells blocked responder cell proliferation and CTL generation. The observation of a CTL response in the H5V system contrasts with previous reports of unsuccessful attempts to generate tumour-specific CTL in polyoma tumour-bearing mice (Dalianis, 1990; Ljunggren et al, 1994). Indeed, the immunogenicity of the middle-T antigen was

© Cancer Research Campaign 1998

only demonstrated after immunization with middle-T peptide fragments, whereas the development of tumour-specific CTL in polyoma tumour-bearing mice was not directly shown (Reinholddson-Ljunggren et al, 1992). A cellular immune response was observed after immunization with irradiated polyoma virus-transformed embryonic fibroblasts; interestingly, high numbers of stimulator cells were also found to block the generation of CTL in this system, although no detailed description of the phenomenon was provided (Green et al, 1982).

The release of soluble immunosuppressive factors by H5V cells in culture provides an explanation for difficulties in demonstrating a CTL response to tumour antigens using high tumour cell numbers as stimulators. We observed that H5V cells produce $PGE_2$ in relatively high quantities; this is not surprising, considering the histological origin of the cell line. Kaposi's sarcoma tissues have also been found to contain high levels of $PGE_2$, which underlines the similarity of the two malignancies (Ambrus et al, 1992). $PGE_2$ may play more than one role in the development of H5V tumours. Its immunosuppressive effects might decrease the efficacy of the anti-tumour cellular immune response; moreover, its angiogenetic properties may contribute directly to the growth of these highly vascularized tumours (Sulitzeanu, 1993; Ben-Av et al, 1995). However, the suppressive activity of different H5V supernatants did not strictly correlate with their $PGE_2$ content; furthermore, blocking of prostaglandin synthesis by indomethacin decreased but did not eliminate the immunosuppressive effect of the supernatants. These results indicate that $PGE_2$ does not represent the only immunosuppressive mediator produced by the H5V cells, and we are currently searching for other suppressive factors in the H5V culture supernatants.

IL-12 had a strong, dose-dependent anti-tumour activity in the H5V tumour model as high doses decreased the size of the primary tumours and prevented tumour regrowth and mortality. It was expected that IL-12 treatment would augment the immune reactivity of the tumour-bearing mice, facilitating tumour rejection. However, the possibility that the in vivo IL-12 treatment acted by increasing the anti-tumour cell-mediated immunity, thus counteracting immunosuppression, seems unlikely, as the CTL and NK activities of the cytokine-treated mice were similar to those of the untreated controls.

In the H5V model, as well as in other tumour models (Brunda et al, 1993; Noguchi et al, 1995; Teicher et al, 1996), relatively high amounts of IL-12 (100 ng–1 μg $day^{-1}$) are necessary for effective tumour elimination, whereas at least 10- to 100-fold lower doses were found to be optimal for boosting the cellular immune reactivity to tumour-specific peptide antigens and to viral or parasite antigens (Biron and Gazzinelli, 1995; Hall, 1995; Noguchi et al, 1995). Therefore, in addition to the lack of an increase in anti-H5V CTL activity after IL-12 treatment both in vitro and in vivo, the discrepancy between the IL-12 dose required to cause tumour regression and boost a cellular immune response in immunization models also suggests that mechanisms other than the increase in cell-mediated immunity might be responsible for the IL-12 effect.

The possibility that IL-12 inhibits H5V tumour cell proliferation directly seems unlikely, as our preliminary experiments showed that IL-12 does not substantially influence H5V proliferation in vitro, as measured by thymidine incorporation (data not shown). Alternatively, IL-12 may inhibit tumour cell proliferation by triggering IFN-γ production by T lymphocytes and NK cells (Gately et al, 1994; Nastala et al, 1994). Our previous studies indicated that IFN-γ suppresses H5V cell proliferation in vitro as well as tumour growth in vivo (Dong et al, 1996). In line with these findings, we observed that anti-IFN-γ MAb co-administration abrogated the anti-tumour effect conferred by IL-12 treatment, thus IFN-γ production is essential for anti-tumour activity of IL-12.

Moreover, IL-12 was shown to suppress corneal neovascularization induced by angiogenic factors, and, like the anti-H5V activity, this antiangiogenetic effect was also IFN-γ-mediated (Voest et al, 1995). The antiangiogenetic effect of IFN-γ is probably the result of the induction of the chemokine IP-10, which was shown to suppress angiogenesis as well as tumour growth in vivo (Tannenbaum et al, 1996). Interestingly, leucocytes attracted by IP-10 seem to be indispensable for its anti-tumour effect (Angiolillo et al, 1995; Mantovani et al, 1997*a,b*; Luster and Leder, 1993).

Based on our findings, therefore, we advance that the anti-tumour effect of IL-12 in H5V tumour-bearing mice is mediated principally by IFN-γ, which not only exerts an inhibitory effect on H5V cell proliferation but probably also on neoangiogenesis. In this regard, it should be stressed that H5V tumours are cavernous haemangiomas consisting of a bulk of recruited host endothelial cells, and a small percentage of true malignant cells, which are also endothelial in origin (Garlanda et al, 1994). Therefore, both the anti-tumour and the antiangiogenetic effects of the IFN-γ induced after IL-12 treatment may contribute to the prevention or regression of this peculiar tumour. However, at present there lacks a methodology to separate these effects. The similarities of the H5V tumour model to Kaposi's sarcoma may open the possibility of using IL-12 to treat Kaposi's sarcoma as well.

### ACKNOWLEDGEMENTS

We thank Dr V Bronte and Dr D D'Agostino for critical reading of the manuscript and V Barbieri for technical assistance. This work was supported by the Associazione Italiana per la Ricerca sul Cancro (AIRC), by Grants from the Italian Ministry of Public Education, by AIDS project (No 9403-68) from the Istituto Superiore Sanità to A Mantovani and by the European Community Project EC-ALA/MED Countries International Scientific Cooperation. C Vizler and L Quintieri are supported by fellowships from Associazione Italiana per la Ricerca sul Cancro. A Rosato is supported by a fellowship from the Istituto Superiore di Sanità – Progetto AIDS.

### REFERENCES

Ambrus JL, Stoll HL, Klein EA, Karakousis CP and Stadler S (1992) Increased prostaglandin E2 and cAMP phosphodiesterase levels in Kaposi's sarcoma – a virus against host defense mechanism. *Res Com Chem Pathol Pharmacol* **78**: 249–252

Angiolillo ALC, Sgadari C, Taub DD, Liao F, Farber JM, Maheswari S, Kleinman H, Reaman GH and Tosato G (1995) Human interferon-inducible protein 10 is a potent inhibitor of angiogenesis in vivo. *J Exp Med* **182**: 155–162

Ben-Av P, Crofford LJ, Wilder RL and Hla T (1995) Induction of vascular endothelial growth factor expression in synovial fibroblasts by prostglandin E and interleukin-1: a potential machanism for inflammatory angiogenesis. *FEBS Lett* **372**: 83–87

Biron CA and Gazzinelli RT (1995) Effects of IL-12 on immune responses to microbial infections: a key mediator in regulating disease outcome. *Curr Opin Immunol* **7**: 485–496

Brunda MJ, Luistro L, Warrier RR, Wright RB, Hubbard BR, Murphy M, Wolf SF and Gately MK (1993) Antitumor and antimetastatic activity of interleukin-12 against murine tumors. *J Exp Med* **178**: 1223–1230

Chieco-Bianchi L, Collavo D and Biasi G (1988) Immunologic unresponsiveness to murine-leukemia-virus antigens: mechanism and role in tumor development. *Adv Cancer Res* **51**: 277–306

Dalianis T (1990) Studies on the polyoma virus tumor-specific transplantation antigen (TSTA). *Adv Cancer Res* **55**: 57–85

© Cancer Research Campaign 1998 

Dong QG, Graziani A, Garlanda C, Wainstok de Calmanovici R, Arese M, Soldi R, Vecchi A, Mantovani A and Bussolino F (1996) Anti-tumor activity of cytokines against opportunistic vascular tumors in mice. *Int J Cancer* **65**: 700–708

Eallarino F, Uyttenhove C, Boon T and Gajewski T (1996) Endogenous IL-12 is necessary for rejection of P815 tumor variants in vivo. *J Immunol* **156**: 1095–1100

Freund R, Dubensky T, Bronson R, Sotnikov A, Carrol L and Benjamin T (1992) Polyoma tumorigenesis in mice: Evidence for dominant resistance and dominant susceptibility genes of the host. *Virology* **191**: 724–731

Garlanda C, Parravicini C, Sironi M, De Rossi M, Wainstok de Calmanovici R, Carozzi F, Bussolino F, Colotta F, Mantovani A and Vecchi A (1994) Progressive growth in immunodeficient mice and host cell recruitment by mouse endothelial cells transformed by polyoma middle-sized T antigen: Implications for the pathogenesis of opportunistic vascular tumors. *Proc Natl Acad Sci USA* **91**: 7291–7295

Gately MK (1993) Interleukin-12: A recently discovered cytokine with potential for enhancing cell-mediated immune responses to tumors. *Cancer Invest* **11**: 500–506

Gately MK, Warrier RR, Honasoge S, Carvajal DM, Faherty DA, Connaughton SE, Anderson TD, Sarmiento U, Hubbard BR and Murphy M (1994) Administration of IL-12 to normal mice enhances cytolytic lymphocyte activity and induces production of IFN-γ in vivo. *Int Immunol* **6**: 157–167

Greene MI, Perry LL, Kinney-Thomas E and Benjamin TL (1982) Specific thymus-derived (T) cell recognition of papova virus-transformed cells. *J Immunol* **128**: 732–736

Hall SS (1995) IL-12 at the crossroads. *Science* **268**: 1432–1434

Jánossy T, Baranyi L, Knulst AC, Vizler C, Benner R, Kelényi G and Végh P (1993) Autoimmunity, hyporeactivity to T cell mitogens and lymphoproliferative disorders following neonatal induction of transplantation tolerance in mice. *Eur J Immunol* **23**: 3011–3020

Katamura K, Shintaku N, Yamaguchi Y, Fukui T, Ohshima Y, Mayumi M and Furusho K (1995) Prostaglandin E2 priming of naive CD4+ T cells inhibits acquisition of ability to produce IFN-γ and Il-12, but not IL-4 and Il-5. *J Immunol* **155**: 4604–4612

Ljunggren G, Ljunggren H-G and Dalianis T (1994) T cell subsets involved in immunity against polyoma virus-induced tumors. *Virology* **198**: 714–716

Luster AD and Leder P (1993) IP-10, a -C-X-C- chemokine, elicits a potent thymus-dependent antitumor response in vivo. *J. Exp. Med.* **178**: 1057–1065

Mantovani A, Bussolino F and Introna M (1997*a*) Endothelial cell activation by cytokines: from molecular level to the bed side. *Immunol Today* **18**: 231–240

Mantovani A, Vecchi A, Sozzana S, Sica A and Allavena P (1997*b*) Tumors as a paradigm for the in vivo role of chemokines in leukocyte recruitment. In *Chemokine and Cancer*, Rollins B (ed), Humana Press: Totova NJ, USA (in press).

Nastala CL, Edington HD, McKinney TG, Tahara H, Nalesnik MA, Brunda MJ, Gately MK, Wolf SF, Schreiber RD, Storkus WJ and Lotze MT (1994) Recombinant IL-12 administration induces tumor regression in association with IFN-γ production. *J Immunol* **153**: 1697–1706

Noguchi Y, Richards EC, Chen YT and Old LJ (1995) Influence of interleukin-12 on P53 peptide vaccination against established Meth A sarcoma. *Proc Natl Acad Sci USA* **94**: 2219–2223

Reinholdsson-Ljunggren G, Ramquist T, Ahrlund-Richter L and Dalianis T (1992) Immunization against polyoma tumors with synthetic peptides derived from the sequences of middle- and large-T antigens. *Int J Cancer* **50**: 142–146

Sciacca FL, Sturzl M, Bussolino F, Sironi M, Brandstetter H, Zietz C, Zhou D, Matteucci C, Peri G, Sozzani S, Benelli R, Arese M, Albini A, Colotta F and Mantovani A (1994) Expression of adhesion molecules, platelet-activating factor, and chemokines by Kaposi's sarcoma cells. *J Immunol* **180**: 4816–4825

Smith WL (1986) Prostaglandin biosynthesis and its compartmentation in vascular smooth muscle and endothelial cells. *Annu Rev Physiol* **48**: 251–262

Sulitzeanu D (1993) Immunosuppressive factors in human cancer. *Adv Cancer Res* **60**: 247–267

Tannenbaum CS, Wicker N, Armstrong D, Tubbs R, Finke J, Bukowski RM and Hamilton TA (1996) Cytokine and chemokine expression in tumors of mice receiving systemic therapy with IL-12. *J Immunol* **156**: 693–659

Teicher BA, Ara G, Manon K and Schaub RG (1996) In vivo studies with interleukin-12 alone and in combination with monocyte colony-stimulating factor and/or fractionated radiation treatment. *Int J Cancer* **65**: 80–84

Trinchieri G (1995) Interleukin-12: a proinflammatory cytokine with immunoregulatory functions that bridge innate resistance and antigen-specific adaptive immunity. *Annu Rev Immunol* **13**: 251–276

van der Pouw Kraan TCTM, Boeije LCM, Smeenk RJT, Wijdens J and Aarden LA (1995) Prostaglandin-E2 is a potent inhibitor of human IL-12 production. *J Exp Med* **181**: 775–779

Voest EE, Kenyon BM, Reilly MSO, Truitt G, Amato RJD and Folkmann J (1995) Inhibition of angiogenesis in vivo by interleukin 12. *J Natl Cancer Inst* **87**: 581–586

© Cancer Research Campaign 1998